# Supplementary material for: Allosteric inhibition of CFTR gating by CFTRinh-172 binding in the pore
Source: Nat Commun. 2024 Aug 6;15:6668. doi: 10.1038/s41467-024-50641-1 (PMC11303713; doi:10.1038/s41467-024-50641-1)
Supplement: Supplementary file 1 — Supplementary Information [file 41467_2024_50641_MOESM1_ESM.pdf]

Supplementary Information for

Allosteric inhibition of CFTR gating by CFTRinh-172 binding in the pore

Xiaolong Gao[1,*], Han-I Yeh[1,2,3], Zhengrong Yang[4], Chen Fan[5,6], Fan Jiang[4], Rebecca J. Howard[5,6], Erik Lindahl[5,6], John C. Kappes[4,7], Tzyh-Chang Hwang[1,2,3,*]

[1]Dalton Cardiovascular Research Center, University of Missouri-Columbia, Columbia, MO 65211, United States.

[2]Institute of Pharmacology, National Yang Ming Chiao Tung University, College of Medicine, Taipei, Taiwan.

[3]Membrane Protein Structural Biology Research Center, National Yang Ming Chiao Tung University, Taipei, Taiwan.

[4]Heersink School of Medicine, University of Alabama School of Medicine, Birmingham, AL 35233, United States.

[5]Department of Applied Physics, Science for Life Laboratory, KTH Royal Institute of Technology, Solna, Sweden.

[6]Department of Biochemistry and Biophysics, Science for Life Laboratory, Stockholm University, Solna, Sweden.

[7]Research Service, Birmingham Veterans Affairs Medical Center, Veterans Health Administration, Birmingham, AL 35233, United States.

[*]Correspondence to Xiaolong Gao (xgdz2@missouri.edu) and Tzyh-Chang Hwang (hwangt@health.missouri.edu)

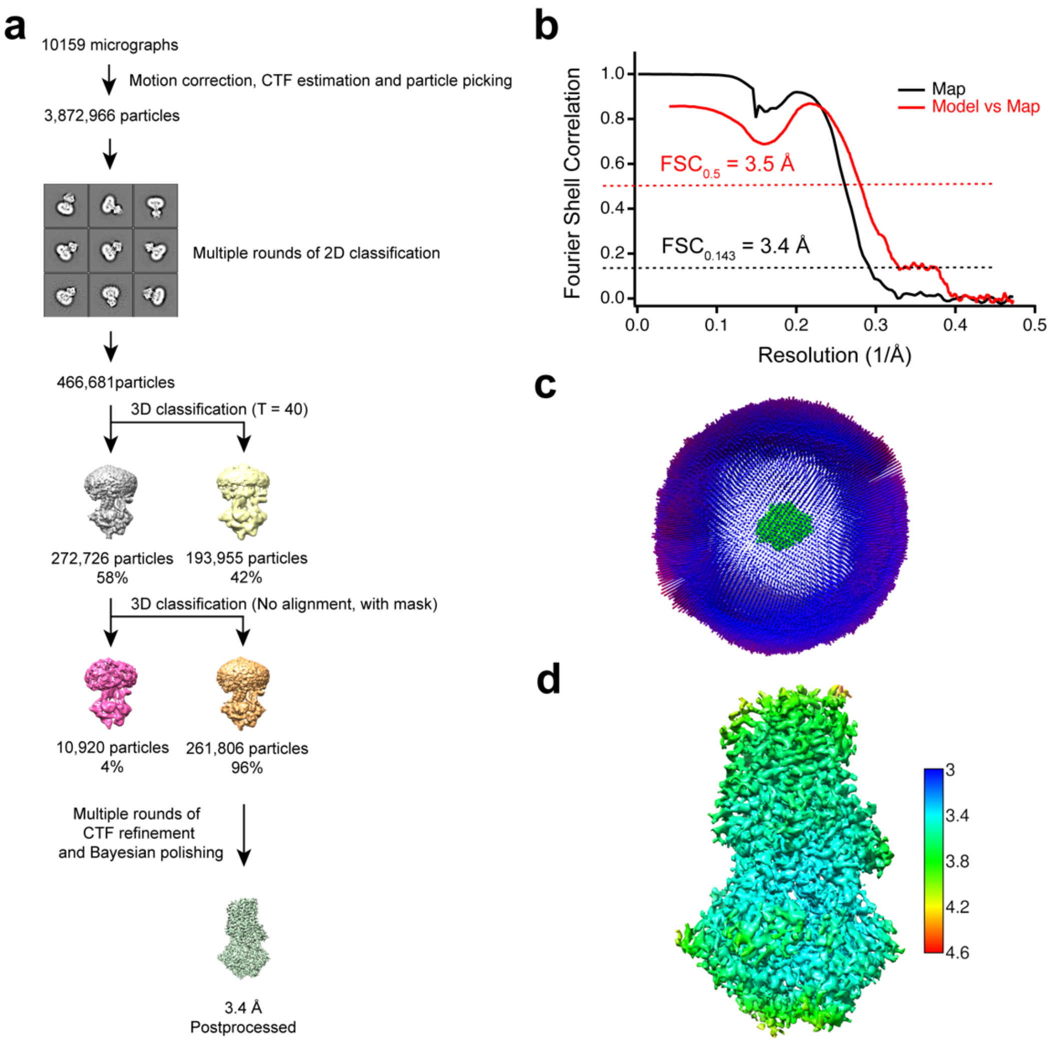

Figure S1. Cryo-EM data processing for E1371Q-CFTR. a Flowchart of E1371Q-CFTR data processing in Relion (see Methods for more details). **b** Fourier Shell Correlation (FSC) curves for E1371Q-CFTR map and model. **c and d** Angle distribution (**c**) and local resolution (**d**) of the final E1371Q-CFTR map.

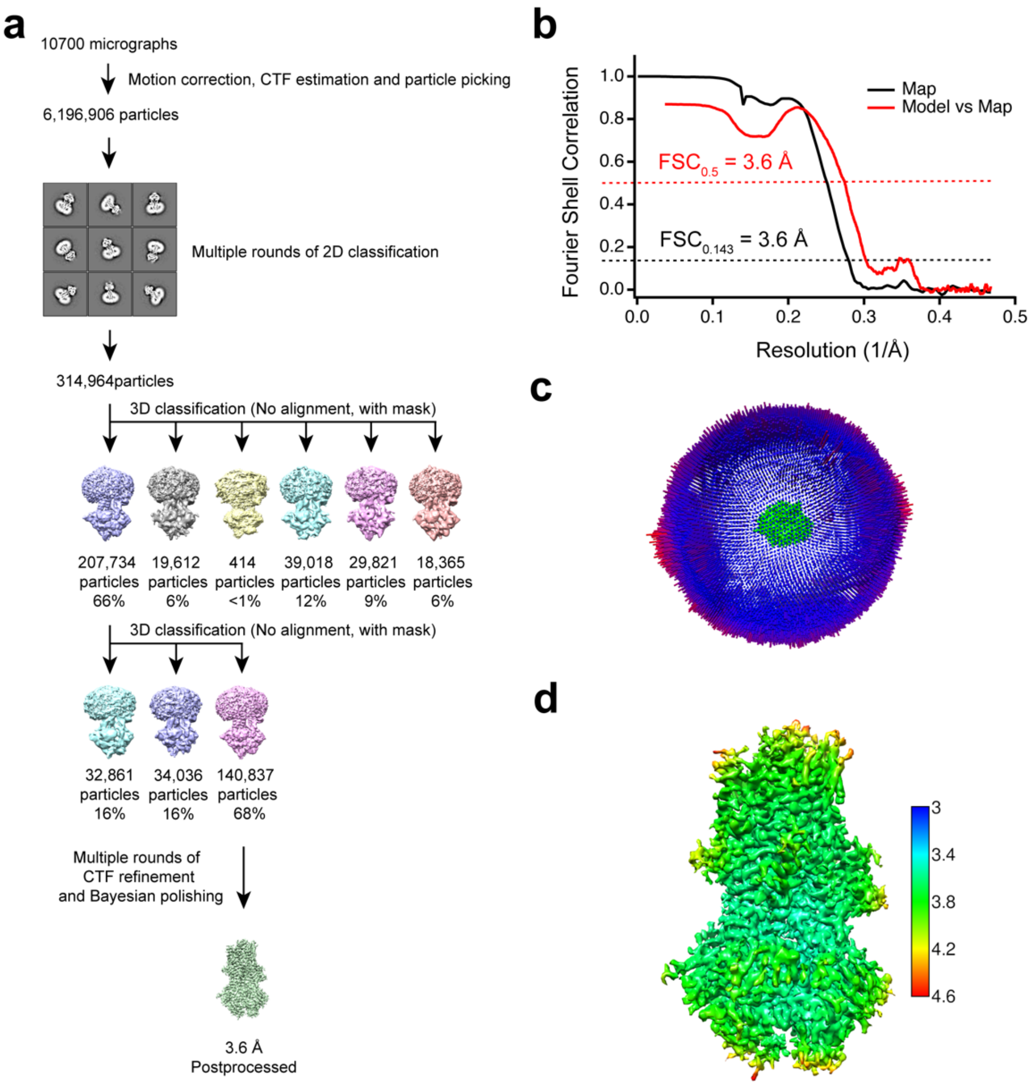

Figure S2. Cryo-EM data processing for Inh-172 bound E1371Q-CFTR. a Flowchart of Inh-172 bound E1371Q-CFTR data processing in Relion (see Methods for more details). **b** FSC curves for Inh-172 bound E1371Q-CFTR map and model. **c** and **d** Angle distribution (**c**) and local resolution (**d**) of the final Inh-172 bound E1371Q-CFTR map.

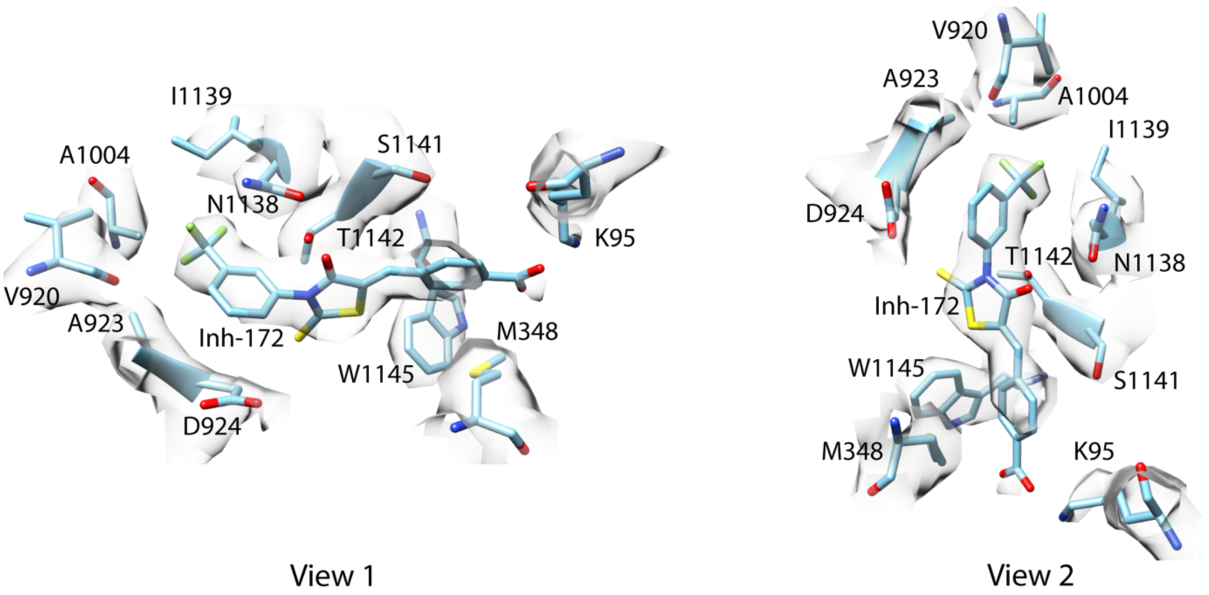

Figure S3. Inh-172 binding site residues in CFTR's pore. Two views showing Inh-172 is coordinated by residues from multiple TMs in the pore. The model is colored based on elements (red, oxygen; blue, nitrogen; yellow, sulfur; green, fluorine and cyan for the backbone). Map density is in light grey.

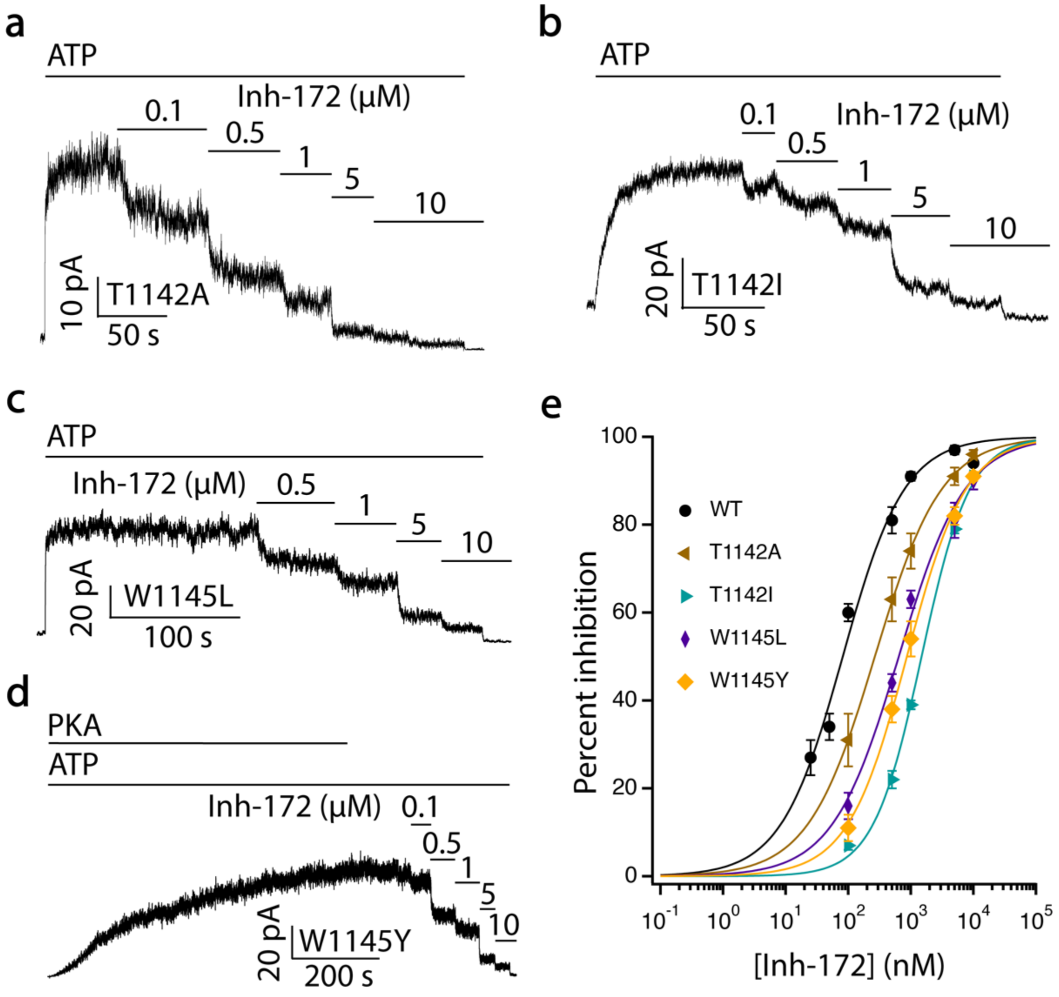

Figure S4. IC_{50} measurements for Inh-172 on binding site mutants. a – d

Electrophysiological experiments showing different degrees of inhibition by Inh-172 at marked concentrations on T1142A-, T1142I-, W1145L-, and W1145Y-CFTR. **e** Percent inhibitions were fitted with equation (2) (see Methods) to obtain the IC_{50} and Hill coefficient values (summarized in Table 1). n = 8, 6, 6, 4, and 5 independent experiments for WT-, T1142A-, T1142I-, W1145L-, and W1145Y-CFTR respectively. Data represent mean ± SEM. Source data are provided as a Source Data file.

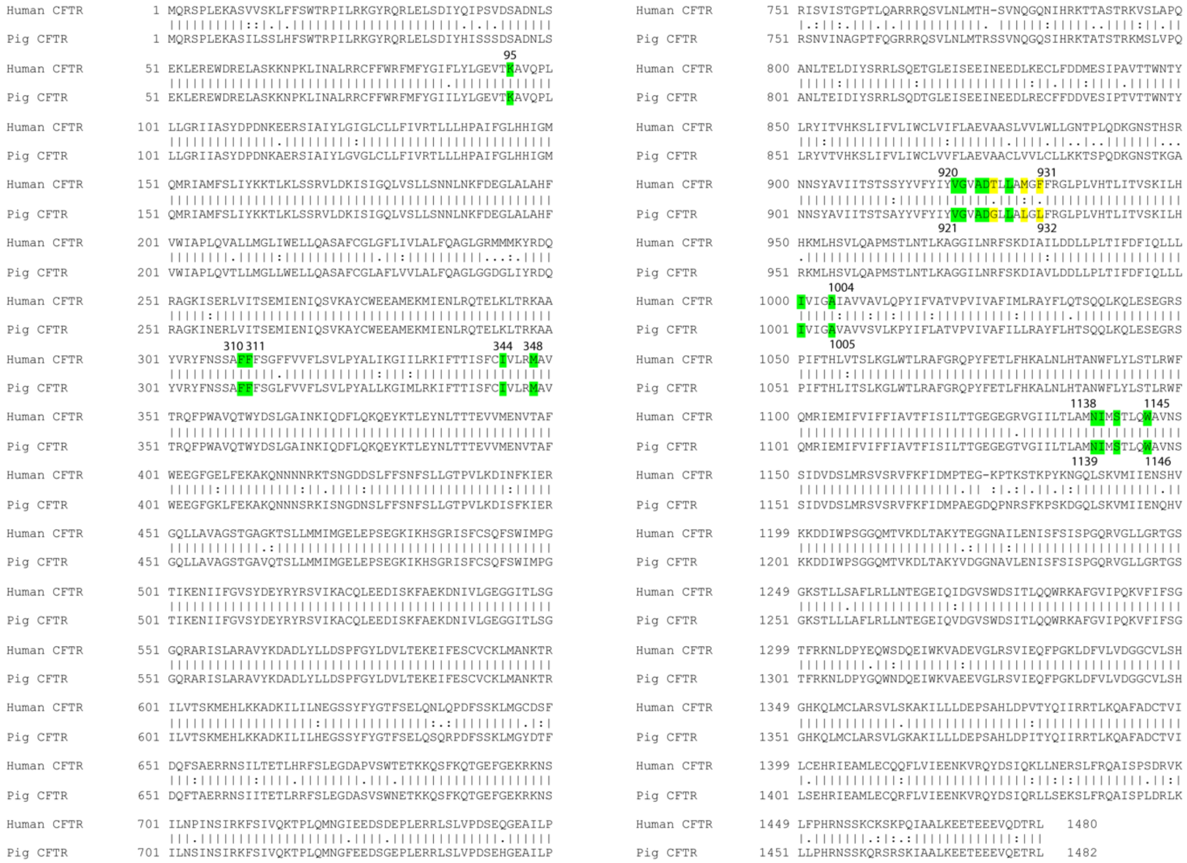

Figure S5. Sequence alignment of human CFTR and pig CFTR. Residues constructing the binding site of Inh-172 in human CFTR and their counterparts in pig CFTR are labeled in green. Note the 100% match for those binding site amino acids. Three residues labeled in yellow mark the differences in the amino acid sequence of TM8 between pig and human CFTR. Vertical lines (|) denote matched residues, periods (.) indicate conservation between groups of weakly similar properties, and colons (:) mean conservation between groups of strongly similar properties.

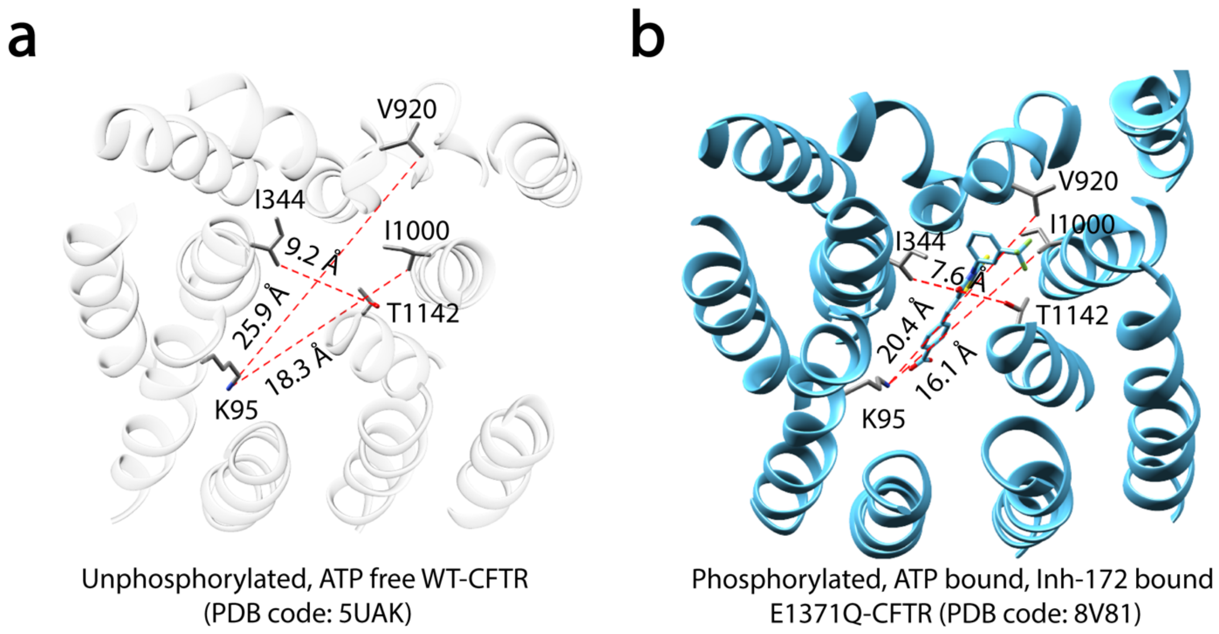

Figure S6. Conformational differences in the Inh-172 binding site between the unphosphorylated, ATP-free state and the phosphorylated, ATP-bound state. a Distances measured among five key residues (dark gray) involved in Inh-172 binding showing the dimension of the binding pocket along or orthogonal to the longitude axis of Inh-172 in the unphosphorylated, ATP-free, WT-CFTR. Three distances marked as red dotted lines were measured. **b** Distances between the same residue pairs in (**a**) were measured for comparison in Inh-172 bound E1371Q-CFTR determined in the current work. Note the orientation of the compound relative to the residue pairs chosen.

Table S1. Data collection, refinement and validation statistics of E1371Q and E1371Q + Inh-172.

	E1371Q<br>EMDB-43011<br>PDB-8V7Z<br>EMPIAR-12042	E1371Q + Inh-172<br>EMDB-43014<br>PDB-8V81<br>EMPIAR-12044
**Data collection and processing**		
Magnification	81000	81000
Voltage (kV)	300	300
Electron exposure (e^-/Å^2)	53.36	65.69
Defocus range (μm)	-0.5 to -2.5	-1 to -1.8
Pixel size (Å)	1.058	1.069
Symmetry imposed	C1	C1
Initial particles images	550,929	314,964
Final particle images	261,806	140,837
Map resolution (Å)	3.4	3.6
FSC threshold	0.143	0.143
**Refinement and validation**		
Initial model used (PDB code)	6MSM	8V7Z
Model resolution (Å)	3.5	3.6
FSC threshold	0.5	0.5
Map sharpening *B* factor (Å^2)	-121	-130
Model composition		
Chains	1	2

Non-hydrogen atoms	8977	9258
Protein residues	1104	1132
Ligands		
POV	5	5
CLR	1	1
ATP	2	2
MG	2	2
LIG		1
B factors (Å^2)		
Protein	66.76	23.43
Ligand	56.24	17.27
Bonds (RMSD)		
Length (Å)	0.002	0.003
Angles (°)	0.585	0.529
Validation		
MolProbity score	1.92	1.57
Clash score	11.72	5.95
Poor rotamers (%)	0.62	0.5
Ramachandran plot		
Favored (%)	95.14	96.34
Allowed (%)	4.86	3.66
Outliers (%)	0	0
Model vs. Data		
CC (mask)	0.72	0.79
CC (box)	0.5	0.63
CC (peaks)	0.52	0.62

CC (volume)	0.69	0.73
