# Supplementary material for: Allosteric inhibition of CFTR gating by CFTRinh-172 binding in the pore
Source: Nat Commun. 2024 Aug 6;15:6668. doi: 10.1038/s41467-024-50641-1 (PMC11303713; doi:10.1038/s41467-024-50641-1)
Supplement: Supplementary file 3 — Reporting Summary [file 41467_2024_50641_MOESM3_ESM.pdf]

nature portfolio

Corresponding author(s): Xiaolong Gao, Tzyh-Chang Hwang

Last updated by author(s): June 18, 2024

Reporting Summary

Nature Portfolio wishes to improve the reproducibility of the work that we publish. This form provides structure for consistency and transparency in reporting. For further information on Nature Portfolio policies, see our Editorial Policies and the Editorial Policy Checklist.

Statistics

For all statistical analyses, confirm that the following items are present in the figure legend, table legend, main text, or Methods section.

n/a	Confirmed	
☐	☒	The exact sample size (n) for each experimental group/condition, given as a discrete number and unit of measurement
☐	☒	A statement on whether measurements were taken from distinct samples or whether the same sample was measured repeatedly
☒	☐	The statistical test(s) used AND whether they are one- or two-sided<br>*Only common tests should be described solely by name; describe more complex techniques in the Methods section.*
☒	☐	A description of all covariates tested
☒	☐	A description of any assumptions or corrections, such as tests of normality and adjustment for multiple comparisons
☐	☒	A full description of the statistical parameters including central tendency (e.g. means) or other basic estimates (e.g. regression coefficient) AND variation (e.g. standard deviation) or associated estimates of uncertainty (e.g. confidence intervals)
☒	☐	For null hypothesis testing, the test statistic (e.g. F, t, r) with confidence intervals, effect sizes, degrees of freedom and P value noted<br>*Give P values as exact values whenever suitable.*
☒	☐	For Bayesian analysis, information on the choice of priors and Markov chain Monte Carlo settings
☒	☐	For hierarchical and complex designs, identification of the appropriate level for tests and full reporting of outcomes
☒	☐	Estimates of effect sizes (e.g. Cohen's d, Pearson's r), indicating how they were calculated

Our web collection on statistics for biologists contains articles on many of the points above.

Software and code

Policy information about availability of computer code

Data collection	Cryo-EM data collection: Leginon<br>Electrophysiology: Pulse 8.53<br>MD simulation: GROMACS 2021.5
Data analysis	Cryo-EM: Relion 4.0, CTFFIND-4.1, Phenix 1.20.1-4487, Coot 0.8.9.2 EL, Chimera 1.17.3<br>Electrophysiology: Igor Pro 8.0<br>MD simulation: MDAnalysis, VMD, ProLIF 1.1.0

For manuscripts utilizing custom algorithms or software that are central to the research but not yet described in published literature, software must be made available to editors and reviewers. We strongly encourage code deposition in a community repository (e.g. GitHub). See the Nature Portfolio guidelines for submitting code & software for further information.

Data

Policy information about availability of data

All manuscripts must include a data availability statement. This statement should provide the following information, where applicable:

- Accession codes, unique identifiers, or web links for publicly available datasets
- A description of any restrictions on data availability
- For clinical datasets or third party data, please ensure that the statement adheres to our policy

The cryo-EM maps and models were deposited into Electron Microscopy Data Bank (EMD-43011 [https://www.ebi.ac.uk/pdbe/entry/emdb/EMD-43011] for E1371Q and EMD-43014 [https://www.ebi.ac.uk/pdbe/entry/emdb/EMD-43014] for Inh-172 bound E1371Q) and Protein Data Bank (8V7Z [https://doi.org/10.2210/pdb8v7z/pdb] for E1371Q and 8V81 [https://doi.org/10.2210/pdb8v81/pdb] for Inh-172 bound E1371Q), respectively. The raw micrographs for both datasets were deposited into Electron Microscopy Public Image Archive (EMPIAR-12042 [https://doi.org/10.6019/EMPIAR-12042] for E1371Q and EMPIAR-12044 [https://doi.org/10.6019/EMPIAR-12044] for Inh-172 bound E1371Q). MD simulation trajectory, parameter files are available in Zenodo (https://doi.org/10.5281/zenodo.10407153). Source data are provided with this paper.

Research involving human participants, their data, or biological material

Policy information about studies with human participants or human data. See also policy information about sex, gender (identity/presentation), and sexual orientation and race, ethnicity and racism.

Reporting on sex and gender	N/A
Reporting on race, ethnicity, or other socially relevant groupings	N/A
Population characteristics	N/A
Recruitment	N/A
Ethics oversight	N/A

Note that full information on the approval of the study protocol must also be provided in the manuscript.

Field-specific reporting

Please select the one below that is the best fit for your research. If you are not sure, read the appropriate sections before making your selection.

☒ Life sciences ☐ Behavioural & social sciences ☐ Ecological, evolutionary & environmental sciences

For a reference copy of the document with all sections, see nature.com/documents/nr-reporting-summary-flat.pdf

Life sciences study design

All studies must disclose on these points even when the disclosure is negative.

Sample size	Predetermination of sample size is not applicable as no statistical analysis is performed. For any experiments that need to be repeated, they will be repeated at least three times.
Data exclusions	Poor quality recordings and recording artifacts are excluded.
Replication	All electrophysiology experiments were repeated on different cells.
Randomization	Randomization is not applicable as our experiments do not require this.
Blinding	Blinding is not applicable as our study does not require this.

Reporting for specific materials, systems and methods

We require information from authors about some types of materials, experimental systems and methods used in many studies. Here, indicate whether each material, system or method listed is relevant to your study. If you are not sure if a list item applies to your research, read the appropriate section before selecting a response.

Materials & experimental systems

n/a	Involved in the study
x	Antibodies
	x Eukaryotic cell lines
x	Palaeontology and archaeology
x	Animals and other organisms
x	Clinical data
x	Dual use research of concern
x	Plants

Methods

n/a	Involved in the study
x	ChIP-seq
x	Flow cytometry
x	MRI-based neuroimaging

Eukaryotic cell lines

Policy information about cell lines and Sex and Gender in Research

Cell line source(s)	Chinese hamster ovary (CHO) cells.
Authentication	The cell line was not authenticated.
Mycoplasma contamination	The cell line was not tested for mycoplasma contamination.
Commonly misidentified lines (See ICLAC register)	No commonly misidentified lines were used in this study.

Plants

Seed stocks	N/A
Novel plant genotypes	N/A
Authentication	N/A
